# “It’s an Uncomfortable Subject”—a Qualitative Exploration of the Challenges and Potential Solutions to Depression Screening in Low Back Pain

**DOI:** 10.1093/ptj/pzaf153

**Published:** 2026-01-07

**Authors:** Julie Sugrue, Sean McKenna, Siobhan MacHale, Kieran O’Sullivan

**Affiliations:** Faculty of Education and Health Science, University of Limerick, Limerick, Co. Limerick, V94T9PX, Ireland; Department of Physiotherapy, Croom Orthopaedic Hospital, Limerick, Co. Limerick, QLJ32KV9, Ireland; Department of Liaison Psychiatry, Beaumont Hospital, Dublin 9, Co. Dublin, D09V2N0, Ireland and Royal College of Surgeons in Ireland, Dublin 2, Co. Dublin, D02YN77, Ireland; Department of Physiotherapy, School of Allied Health, University of Limerick, Limerick, Co. Limerick, V94T9PX, Ireland

**Keywords:** Depression, Low back pain, Physical therapists

## Abstract

**Importance:**

Comorbid depression in people with low back pain (LBP) is associated with poorer prognosis.

**Objective:**

The objective was to understand the challenges faced by musculoskeletal (MSK) triage physical therapists when screening for depression in LBP populations, and to generate actionable recommendations for overcoming these challenges.

**Design:**

This study adopted a pragmatic hybrid descriptive qualitative approach, integrating elements of ethnography and action research.

**Setting:**

Interviews were conducted in the Republic of Ireland and used purposive sampling of physical therapists working in MSK triage roles.

**Participants:**

To be included, participants were required to have managed at least 1 person with LBP each week in the 3 months prior to recruitment.

**Intervention(s) or Exposure(s):**

The context explored was MSK triage physical therapists’ experience with depression screening in people with LBP.

**Main Outcomes and Measure(s):**

The main outcomes were insights regarding challenges and potential solutions to depression screening. Semi-structured interviews were employed, with data analysis following a Reflexive Thematic Analysis framework.

**Results:**

Fourteen MSK triage physical therapists participated. Challenges were organized into 3 themes: capacity (personal*,* professional*,* and system), culture (clinic*,* societal), and circuitous communication. Potential solutions were organized into 5 themes: training and education, standardized pathways, knowledge of and access to resources, screening tools, and normalizing depression screening in MSK triage equivalent to red flag screening.

**Conclusion:**

The findings highlight capacity and cultural challenges that lead to circuitous communication. Addressing the potential solutions through implementation research could enhance depression screening practices by MSK triage physical therapists for people with LBP.

**Relevance:**

This qualitative research offers novel insights into the challenges MSK triage physical therapists face when screening for depression in people with LBP. Importantly, it proposes actionable solutions with participants contributing as subject matter experts. Their pragmatic solutions can help facilitate consequential change and help normalize depression screening in MSK triage practice.

## INTRODUCTION

Low back pain (LBP) remains the leading cause of disability worldwide and contributes to the highest burden among musculoskeletal (MSK) disorders.^1^ The presence of comorbid depression further amplifies this disability and is associated with poorer outcomes.^2^ The biopsychosocial model of LBP management emphasizes the early identification and management of psychosocial risk factors, commonly referred to as yellow flags, to prevent chronicity and improve prognosis.^3–5^ Several multidimensional screening tools such as the Örebro Musculoskeletal Pain Questionnaire,^6–8^ the STarT Back,^9^ and the Optimal Screening for Prediction of Referral and Outcome^10^ are available to assess these factors. Although these tools include items related to depressive symptoms, they were developed to identify the risk of chronicity rather than to identify clinical depression requiring onward referral. Despite their availability, such tools remain underutilized in LBP assessment,^11^^,^^12^ with physical therapists frequently relying on subjective clinical judgment^12^ and often overestimating their ability to detect psychosocial issues without structured support.^13^

In contrast to yellow flags, orange flags refer to mental health conditions requiring psychological and/or psychiatric intervention, such as clinical depression, suicidal ideation, or major mental illness.^4^^,^^14^ As depressive symptoms and clinical depression are increasingly understood not to be discrete categories,^15^ it may be more appropriate to conceptualize yellow and orange flags as parts of a continuum.^16^ Although formal diagnosis of psychopathology lies outside physical therapy’s scope of practice, screening for and onward referral of orange flags, such as depression, is widely recognized as a professional responsibility.^16^^,^^17^ However, physical therapists often perceive their role as limited to pain-related distress,^16^ and report feeling unprepared to manage depression or suicidal ideation.^18^ While some LBP guidelines recommend depression screening and onward referral as required, few offer clear or practical implementation strategies.^14^ Given the strong association between depression and poorer LBP outcomes, including greater disability and delayed recovery,^2^ early identification and treatment of depression is critical to optimizing LBP care.^19^ The urgency of addressing depression in LBP care is further highlighted by evidence that the lifetime prevalence of suicide attempts among people with major depressive disorder is 31%,^20^ and that over 20% of people who died by suicide had a history of hospital-treated MSK disorders.^21^

Physical therapists working in first contact practitioner roles act as gatekeepers to MSK secondary care services for people with LBP across Australia, Canada, New Zealand, Ireland, and the United Kingdom. They are expected to adopt a holistic, biopsychosocial approach when managing LBP, including screening for red, orange, and yellow flags to guide appropriate and timely care.^4^ Evidence indicates that physical therapists’ clinical impression alone is unreliable for detecting depression in people with spinal pain.^22–25^ A recent scoping review^26^ recommends the deliberate use of direct screening questions or validated tools to improve identification. A follow-up survey of Irish MSK triage physical therapists^27^ found that only 8% routinely screened directly for depression in people with LBP, with lack of training and uncertainty about managing disclosures cited as barriers. That study^27^ offered a valuable snapshot of current screening practices and compared confidence levels between red, yellow, and orange flag screening. This study builds on those findings through in-depth qualitative interviews with a subset of the same Irish MSK triage physical therapy cohort.

The primary aim of this study is to explore the factors contributing to low rates of depression screening in people with LBP by MSK triage physical therapists. A secondary aim is to identify practical solutions that might address these challenges. The overall objective is to understand the challenges MSK triage physical therapists face in screening for depression in LBP populations and to generate actionable, context-specific recommendations to support clinical practice.

## METHODS

### Design

This study employed a pragmatic, hybrid descriptive qualitative approach, integrating elements of ethnography and action research.^28^ Semi-structured interviews explored MSK triage physical therapists’ experiences and perspectives. Grounded in a pragmatic ontology, the study sought to generate practical insights closely aligned with participants’ real-world experiences, without relying on a predefined theoretical framework. Incorporating ethnographic and action research components provided contextual depth, illuminating how depression screening is navigated in everyday clinical practice.

Data were analyzed using Braun and Clarke’s Reflexive Thematic Analysis (RTA), a flexible, inductive, and iterative method aligned with the study’s philosophical stance.^29–31^ Reporting followed the Standards for Reporting Qualitative Research^32^ checklist (Suppl. Material 1).

### Participants and Context

Ethical approval was granted by the University of Limerick Faculty of Education and Health Sciences Research Ethics Committee (2024_02-06_EHS). Participants were recruited via purposive sampling. An invitation to complete an anonymous survey, with an option to participate in follow-up interviews, was emailed to gatekeepers such as public hospital managers and peer professional network groups across the Republic of Ireland, requesting circulation to all physical therapists working in MSK triage roles. The eligible sample, excluding those on long-term leave of absence or not accepting LBP referrals, comprised 65 MSK triage physical therapists. Of these, the 36 survey participants^27^ were prompted to provide their email address if they wished to take part in interviews. Inclusion criteria required participants to have managed at least 1 person with LBP per week in the prior 3 months and to have provided written informed consent.

### Interview Schedule and Procedures

The interview guide (Suppl. Material 2) was designed to align with the study’s aims, balancing core research topics with flexibility to allow participant-led dialog. Face validity was established through review by 3 former MSK triage physical therapists, a consultant liaison psychiatrist, and 2 clinical researchers. Interviews were conducted via Microsoft Teams (Microsoft Corp) to enhance accessibility and facilitate transcription. Participants joined from home or work settings; 1 interview experienced brief interruptions.

Semi-structured, open-ended questions allowed flexible exploration of topics, encouraging elaboration without unnecessarily extending interview duration. Participants reflected on their experiences with depression screening and onward referral in LBP, as well as potential solutions. Field notes were recorded post-interview. Transcripts were corrected and pseudonymized, and were not returned to participants for member checking. Basic demographic data (eg, sex, years of experience) were collected to provide contextual information.

### Data Analysis

Data analysis was guided by Braun and Clarke’s 6 phases of RTA^29–31^: (1) familiarization, (2) initial coding, (3) theme development, (4) reviewing themes, (5) defining and naming themes, and (6) reporting (Suppl. Material 3). This approach facilitated an in-depth exploration of participants’ beliefs and experiences, aligning with the study’s descriptive focus without imposing a broader methodological framework.

The lead author (J.S.) conducted primary analysis, with fortnightly discussions with a second researcher (K.O.S.) to enhance rigor. NVivo qualitative data analysis software (Version 14; QSR International; Melbourne, Australia) was used to organize data, while theme development remained a human reflexive and interpretive task.

### Researcher Characteristics and Reflexivity

The lead author (J.S.) is a White female MSK triage physical therapist with 20 years’ MSK experience. Her clinical background informed a practice-oriented perspective. Situated within a pragmatic qualitative paradigm, her insider perspective enhanced contextual understanding. Reflexivity was maintained through regular reflection on assumptions and positionality. For example, an initial assumption that participants would feel unprepared for managing suicidal disclosures was not supported by the data. These reflections were documented to support transparency. The wider research team included a second MSK triage physical therapist researcher (S.McK.), a consultant liaison psychiatrist (S.McH.), and a senior physical therapy researcher (K.O.S.), whose diverse expertise enriched interpretation.

#### Trustworthiness

Trustworthiness was supported through deep engagement with the data, transparency, and reflexivity consistent with RTA principles.^29–31^ Strategies included prolonged immersion, regular analytic discussions, and maintenance of an audit trail (Suppl. Material 3). Participant quotes throughout the results section support authenticity. The semi-structured, participant-led interviews reflected the study’s pragmatic orientation. All interviews covered the guide comprehensively and contributed meaningfully to theme development.

## RESULTS

Fourteen participants consented to taking part in qualitative interviews, conducted between April and July 2024. There were no dropouts. Interview duration ranged from 16 to 39 minutes, with a mean length of 26 minutes. Despite 1 interview being relatively brief, all interviews addressed the core questions. Participants were predominantly female (64%) with a mean age of 42 years (SD = 3.9), and had been working in MSK triage roles for a mean of 5 years (Table 1).

**Table 1:** Characteristics of Participants (*n* = 14)*^a^*

Pseudonym	Sex	Years Qualified	Years in MSK Triage	Formal Training^*b*^	Informal Training^*c*^	Suicide Training^*d*^	Primary Work Setting (Clinic Type)
David	M	21	8	>1 d	>1 d	No	Public Hospital Outpatients (Ortho/Rheum)
Alice	F	14	3	<1 d	>1 d	No	Public Hospital Outpatients (Ortho/Rheum)
John	M	14	8	None	<1 d	No	Public Hospital Outpatients (Ortho)
James	M	18	2	None	<1 d	No	Public Hospital Outpatients (Ortho)
Daniel	M	10	2	None	>1 d	Yes	Public Hospital Outpatients (Ortho/Rheum)
Anna	F	16	1	>1 d	>1 d	Yes	Public Hospital Outpatients (Pain)
Emily	F	23	15	>1 d	>1 d	No	Public Hospital Outpatients (Ortho/Rheum)
Sophie	F	23	6	None	None	Yes	Public Hospital Outpatients (Ortho)
Grace	F	21	9	None	>1 d	No	Public Hospital Outpatients (Ortho/Rheum)
Emma	F	17	2	None	<1 d	Yes	Public Hospital Outpatients (Ortho/Rheum)
Kate	F	23	2	None	<1 d	Yes	Public Hospital Outpatients (Ortho)
Lisa	F	18	5	<1 d	>1 d	Yes	Public Hospital Outpatients (Ortho)
Leah	F	12	1	<1 d	>1 d	Yes	Public Hospital Outpatients (Pain)
Michael	M	16	11	None	<1 d	Yes	Public Hospital Outpatients (Ortho)

^a^ Abbreviations: d = day; MSK = musculoskeletal; Ortho = orthopedic; Rheum = rheumatology.

^b^ Formal Training = Completed formal training on screening for depression (eg, postgraduate course).

^c^ Informal training = Completed informal training on screening for depression (eg, peer support).

^d^ Suicide Course = Completed specific training on suicide awareness (eg, Safe Talk).

### Challenges to Screening for Depression in MSK Triage

**Figure 1:**
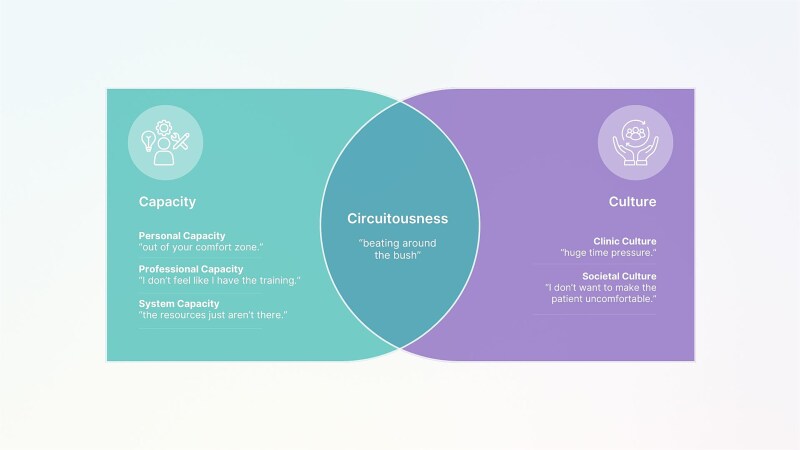
Challenges to Depression Screening in Musculoskeletal Triage.

Participants described multiple challenges to routinely screening for depression in MSK triage, which clustered around 3 overarching themes: capacity, culture, and circuitousness. Capacity and culture were organized into subthemes, with the interplay between these leading participants to adopt a circuitous communication style, rather than asking directly about depression (see Table 2, Figure 1, and Suppl. Material 4).

**Table 2:** Challenges to Depression Screening in Musculoskeletal Triage*^a^*

Theme / Subtheme	Illustrative Participant Quotations
**Capacity “*How Are You Going to Deal With [It]”***	
**Personal capacity *“Out of Your Comfort Zone”***	*“There’s safety in not knowing stuff. If you don’t ask, you don’t know, so you don’t have to explore that…it’s a protective thing and like, it is scary. Like when patients are talking about, ‘I was sitting at home with a bottle of pills, a bottle of pills and a bottle of whiskey the other day,’ and you’re asking him, like ‘do you have a plan?,’ like, that’s terrifying… and patients that will tell you I’ve had 4/4 suicide attempts and you’re like ‘OK. Any plans currently?’ Which is terrifying”* (James) *“It’s when you feel that you’re going out of your comfort. It’s when you’re out of your comfort zone…out of your kind of realms of professional, I suppose capacity as well, like, that’s when it just, that’s where you feel like if you had back up, you just want to feel that you have back up”* (Kate) *“There is always the fear of the, fear of the unknown. What are you going to uncover? What are they going to say? How are you going to deal with whatever is disclosed to you, I suppose….[you’re] almost nervous having the conversation because you’re afraid of what you’re going to unearth” (Kate)*
**Professional capacity** ***“I Don’t Feel Like I Have the Training”***	*“I don’t feel like I have the training to even go there like, so I don’t know if I’d ever ask someone do you have depression”* (Grace) *“The hard part is knowing where to direct patients…I don’t feel I have the expertise or the knowledge to know where the most appropriate person or the most appropriate place to start care is… we have a problem with knowing what to do with the answer”* (Emma) *“When does someone meet a threshold? Do you know what I mean? Yeah. Yeah, it is tricky. It is tricky….I wouldn’t routinely screen for a reason being that, yeah, maybe I don’t feel like I have the tools to act on signs”* (Michael)
** System capacity** **“The Resources Just Aren’t There*”***	*“I think I think you have to be careful because when you delve into something, you have to be able to provide a solution for it”* (Alice) *“The thing that I think we lack is that talk therapy component to working with any sort of mental health unless someone has serious mental health problems, the resources just aren’t there”* (James) *“I think it is under resourced massively throughout the Community”* (Anna)
** Culture *“It’s Difficult”***	
** Clinic culture** **“*Huge Time Pressure*”**	*“There’s a huge time pressure in our role… I have to figure out if there’s any other red flags, and what I should do with them, if they need imaging, or injections, or physiotherapy, and it’s really challenging to do all that and figure out if there is depression in a short triage appointment”* (David) *“You’ve got a certain amount of time and you need to do a certain amount of physical tests at a patient and rule out anything sinister, and that squeezes the time then for the kind of more lengthy questions which are more the psychosocial side of things”* (Alice) *“A 15–20 minute consultation, it’s really, you know, you might you might cover it if someone you know, expresses a desire to cover, but otherwise you’ll… get through the absolute bare minimum”* (Michael)
**Societal culture** ***“I Don’t Want to Make the Patient Uncomfortable”***	*“It’s an uncomfortable subject for a lot of people to talk about whether they’re depressed or not. And that’s a cultural thing. Like Irish people aren’t necessarily open about talking about mental health”* (James) *“I think there’s something about ‘are you depressed?’ I’m not sure that people would, would necessarily, tell you….maybe it’s my anticipating that people won’t want to admit to it”* (Sophie) *“I don’t want to make the patient uncomfortable”* (Grace)
**Circuitousness**	
**“*Beating Around the Bush”***	*“I would definitely ask every single person, but possibly not specifically start with depression as a question, but more ask them around their mood and how their back pain’s affecting their mood and from whatever comes up from that, see where we go*” (James) *“I probably wouldn’t ask outright often. Are you depressed? I might talk about low mood. And do you think mood is, you know, influencing your symptoms or influencing your pain and see if I can get them to open up a little bit that way about it… start with the more functional or social kind of engagement, or umm, hobbies and whatnot, and then it’s sometimes a bit easier to lead into other parts of their life…we have been beating around the bush like I was describing”* (Sophie) *I’d probably go indirectly. I would go indirectly. There is discomfort there. Some patients more than others. So you’ve just feel a little bit more comfortable probing it or discussing it I suppose. But yeah, there is always the fear of the unknown. What are you going to uncover? What are they going to say? How are you going to deal with whatever is disclosed to you, I suppose?...I think it’s not something that you can just go like head straight first into kind of. Maybe you should…I probably more so react going with prompts from the patient…tiptoeing around it”* (Kate)

^a^ See also Supplementary Material 4**.**

#### Capacity “How Are You Going to Deal With [It]”

Capacity challenges were organized into personal, professional, and system capacity subthemes. Across these, participants reported feeling uneasy, ill-equipped, and unsupported when screening for depression.

#### Personal Capacity “Out of Your Comfort Zone”

Participants spoke about personal discomfort when triage conversations turned towards depression. Screening was seen as moving beyond the safety of MSK concerns into unpredictable territory. Several described fear—fear of the unknown, of managing disclosures, or of starting conversations they might not know how to close.

Many acknowledged the importance of asking, but felt challenged by being out of their “comfort zone” (Kate). For some, avoidance became a coping strategy. One participant admitted there was a “safety in not knowing” (James), while others openly questioned whether depression screening was part of their role. These reflections highlight how personal discomfort can overlap with questions of professional identity.

#### Professional Capacity “I Don’t Feel Like I Have the Training”

Beyond personal discomfort, participants described feeling under-equipped due to limited training, knowledge, and skills. Some reflected on not having “the training to even go there” (Grace) when they considered depression screening, while others expressed uncertainty in the interpretation of responses and when to escalate concerns.

Participants expressed greater confidence in managing active suicidal ideation than depression without suicidality, reflecting relatively clear crisis management processes. Limited knowledge and a lack of guidance regarding services and referral options for people with depression without active suicidal ideation so that participants had little clarity on “what to do with the answer” (Emma) contributed to screening hesitation. Many defaulted decision-making to the primary care physician, reinforcing the perception that depression screening falls outside scope of MSK triage physical therapy.

#### System Capacity “The Resources Just Aren’t There”

Even when participants were prepared to screen for depression, many doubted whether community mental health services could provide meaningful follow-up. Several emphasized that “when you delve into something, you have to be able to provide a solution for it” (Alice), but their experience suggested the system was not capable of absorbing additional referrals.

A perception of under-resourced services prevailed. Participants were reluctant to identify depression when they knew “the resources just aren’t there” (James). Uncertainty about eligibility, referral rights, and waiting times further reinforced distrust in the system and left the MSK triage physical therapists feeling isolated, vulnerable, and unsupported.

#### Culture “It’s Difficult”

Culture challenges were organized into clinic and societal subthemes, reflecting how the work environment and broader societal attitudes toward depression influenced screening.

#### Clinic Culture “Huge Time Pressure”

Within the MSK triage setting, participants described a culture of intense “time pressure” (David) that left little space for open-ended and unpredictable depression screening. Appointments of 15 to 20 minutes were already packed with the biomedical tasks of ruling out red flags, ordering imaging, and providing initial management. Screening for depression was seen as a time-consuming task requiring “more lengthy questions” (Alice).

Participants admitted to skirting around depression screening, acknowledging tension between holistic care and a fast-paced triage model, which prioritizes efficiency and throughput. Participants recognized the importance of psychological care in LBP management but felt the triage structure was inherently incompatible, with frustration adding to the emotional toll of an already pressured role.

#### Societal Culture “I Don’t Want to Make the Patient Uncomfortable”

Participants viewed depression as a topic that patients might be reluctant to discuss. Although participants themselves did not express stigma, they anticipated patient resistance, assuming direct questioning would make patients feel judged. One participant noted “I don’t want to make the patient uncomfortable” (Grace). Others acknowledged this reluctance often stemmed from their own “anticipating that people won’t want to admit to it” (Sophie) rather than patients’ actual reactions.

Societal norms positioning depression as a private matter contributed to the perception that screening was intrusive unless patient-initiated. These MSK triage physical therapists valued patient-centered care and recognized the importance of depression screening for LBP outcomes but remained cautious about overstepping social norms.

#### Circuitousness “Beating Around the Bush”

Intersecting capacity and culture was a circuitous style of communicating, whereby participants frequently relied on indirect ways of addressing depression, preferring subtle cues, vague language, or waiting for patients to raise the issue. Several described “tiptoeing around” (Kate) or “beating around the bush” (Sophie), asking how “back pain is affecting their mood” (James) rather than naming depression directly.

At times, circuitousness was used as a coping strategy against personal discomfort associated with fear of what questioning might unearth. Without confidence in their ability to screen or respond appropriately, indirect approaches were seen as a safer way to acknowledge psychological factors and distress without triggering uncomfortable discussions. Limited professional training and lack of clear guidance contributed to avoidance of direct questioning. This was reinforced by the perceived futility of inaccessible and under-resourced follow-up services.

Clinical culture shaped this further, with lengthy open-ended conversations threatening to derail tightly controlled triage time slots. Indirect phrasing was also seen as more acceptable to perceived societal norms that depression is a private or sensitive matter, and that patients might feel judged or uncomfortable if screened directly.

Overall, circuitousness allowed participants to acknowledge depression without fully engaging with it, reducing discomfort and uncertainty and maintaining efficiency, likely with opportunities for timely identification and support missed as a consequence.

### Potential Solutions to Depression Screening in MSK Triage

**Figure 2:**
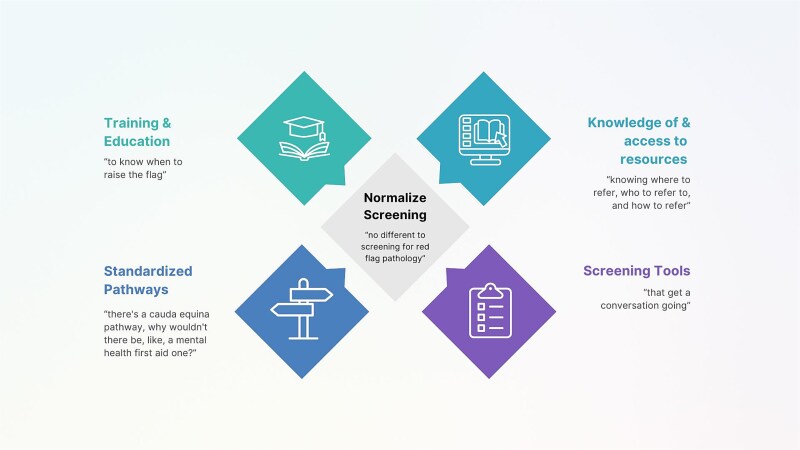
Solutions to Depression Screening in Musculoskeletal Triage.

Participants identified 5 potential solutions to support more effective and consistent depression screening in MSK triage: training and education, standardized pathways, knowledge of and access to resources, pragmatic screening tools, and normalization of depression screening (see Figure 2 and Suppl. Material 5).

#### Training and Education “To Know When to Raise the Flag”

All participants emphasized that training is essential to equip MSK triage physical therapists to confidently identify and respond to depression, ensuring that “everybody’s brought up to a standard” (James) to be able “to ask the questions and have an idea what to do with the given response” (John). Training was envisioned as a core component of professional development alongside essential clinical skills and mandatory education.

Participants highlighted the value of multiple perspectives from a variety of experts including psychiatrists, psychologists, psychotherapists, and patients, as well as informal peer learning. Preferences for delivery varied as some favored concise online modules for accessibility, while others advocated for face-to-face workshops or day-long sessions that allow interaction, reflection, and practical application. Regardless of format, participants underscored the need for practical and targeted training that provides actionable guidance and builds knowledge and confidence so that they can initiate and manage conversations about depression.

#### Standardized Pathways “There’s a Cauda Equina Pathway. Why Wouldn’t There Be, Like, a Mental Health First Aid One?”

Participants highlighted the importance of clear, standardized referral pathways to support confident depression screening. Several emphasized that having “really clear pathways” (Alice) and would enable them to act decisively. The absence of structured pathways was contrasted with established systems for physical emergencies: “there’s a cauda equina pathway, why wouldn’t there be, like, a mental health first aid one?” (Leah).

Structured pathways would guide predictable, stepwise action when depressive symptoms are identified, with their clarity and decisiveness likened to fire drills: “in case of emergency break glass” (James). The relative confidence participants expressed in managing crisis situations such as active suicidal ideation, which was seen as “a little bit more straightforward” (Sophie) due to well-defined required actions, illustrated how standardized pathways could extend that confidence to more ambiguous depression screening. Developing and embedding such pathways was viewed as a practical step towards ensuring depression screening leads to appropriate and timely support.

#### Knowledge of and Access to Resources “Knowing Where to Refer, Who to Refer to, and How to Refer”

While recognizing that community services are “under resourced and under pressure” (David), participants identified improved knowledge and access to mental health resources as essential to ensuring depression screening translates into timely support. Knowing that appropriate supports were in place would give them assurance to “open that can of worms” (Alice) with patients. They emphasized the value of clearly defined referral options so that they would know “where to refer, who to refer to, and how to refer” (Kate). Increasing awareness of local counseling, psychology, and psychiatric services would enable clinicians to act confidently and follow through after screening, rather than defaulting to referral back to the primary care physician or leaving patients “in limbo” (James).

Participants did not view asking about depression as the main difficulty, rather, as 1 explained “we have a problem with knowing what to do with the answer and if we had that knowledge” (Emma). The availability of clear referral criteria and accessible resources was viewed as crucial to facilitating confident, effective, and meaningful depression screening in LBP care.

#### Screening Tools “That Get a Conversation Going”

Participants highlighted how brief, user-friendly, and pragmatic screening tools could serve as prompts to initiative conversations about depression and to guide clinical judgment. One participant described the ideal as “a tool that you can either formally do with the patient or that you can kind of nearly mark off yourself based on that conversation” (Kate). Validated tools with reasonable sensitivity were seen a way to increase confidence and ensure consistency. By providing both structure and language for what can otherwise feel like an uncertain and sensitive topic, brief validated tools were viewed as conversations starters that can support the integration of depression screening into routine LBP care.

#### Normalize Screening “No Different to Screening for Red Flag Pathology”

Participants emphasized that depression screening should be “no different to screening for red flag pathology” (John)*.* Embedding it within established assessment frameworks, so that it becomes “part of your standardized objective” (James) was seen as key to achieving this consistency, and removing uncertainty and stigma from the process.

Normalization was described not only as a cultural shift, but as a procedural 1, achievable through consistent habit: “if you just make it a normal part of your process then…the worst thing is they say I’m fine” (Anna). Achieving this normalization in MSK triage is likely to require the coordinated implementation of brief, pragmatic screening tools to support conversations, training and education to build confidence, and structured pathways and resources to facilitate appropriate onward care.

## DISCUSSION

This study identified interrelated capacity and cultural challenges that limited depression screening by MSK triage physical therapists for people with LBP, leading to indirect and circuitous screening approaches. Despite these challenges, participants proposed clear and actionable recommendations to enhance depression screening practices in LBP care. These pragmatic solutions focused on targeted training and education, standardized pathways, access to appropriate services and resources, and pragmatic screening tools. Ultimately, participants emphasized the importance of normalizing depression screening as a routine component of LBP assessment in MSK triage practice, so that it becomes no different from red flag screening.

Participants expressed greater confidence in managing active suicidal ideation than depression without active suicidal ideation, reflecting confidence in established crisis procedures. This may also reflect a training effect, as half of participants had completed suicide-awareness training. The framework of non-MSK flags proposed by Stearns et al,^18^ similarly depicts active suicidal ideation as a red flag. While previous studies have highlighted physical therapists’ hesitancy in addressing suicidal ideation,^33–35^ the current findings suggest that procedural clarity fosters confidence and decisive action. In contrast, depression screening remains more ambiguous, requiring greater interpretation of symptoms and decisions, leaving clinicians more vulnerable to the challenges described by participants.

Personal, professional, and system capacity constraints emerged as central challenges to depression screening. Emotional discomfort, a personal capacity constraint, appeared to contribute to the avoidance of direct questioning. This aligns with literature on topic avoidance,^36^ where physical therapists may redirect conversations towards physical issues to maintain emotional distance.^37^ Responding to patients’ emotions while regulating one’s own can be stressful,^38^ even though clinician vulnerability is key for effective consultations.^39^ Previous research suggests that the practice of waiting for the patient to raise psychological concerns can serve to relieve physical therapists of responsibility for initiating such conversations.^40^

Professional capacity gaps, particularly in training and referral knowledge, further limited participant’s ability to screen effectively. Many reported uncertainties about which questions to ask, how to interpret responses, and where to refer patients, reflecting findings from previous research.^33^^,^^35–37^^,^^41–45^ To address these gaps, participants recommended interprofessional training that includes patient involvement. As McGrath et al^16^ emphasize, training must integrate both technical and emotional components to support confident decision-making and help physical therapists navigate uncomfortable conversations. A hybrid training model, combining online modules for foundational knowledge with in-person workshops for skills practice and reflective discussion, would accommodate varied preferences.

Most participants had received some form of depression screening training, with a prior survey finding that this was primarily informal and self-directed.^27^ Such training appears insufficient to build lasting confidence or competence, consistent with evidence that short-term or single-session skills training rarely translates into sustained practice change or improved outcomes.^46^ Revising academic curricula to include mental health training is essential,^47^ alongside clear professional scope-of-practice guidelines and supportive organizational policies to embed screening into routine LBP care.^43^^,^^48^^,^^49^

The absence of professional policy and guidance addressing comorbid mental health screening likely contributes to uncertainty about roles.^50^ However, evolving policy provides a foundation for change. Statements from the International Organization of Physical Therapists in Mental Health^51^ articulate the scope of physical therapists within mental health care, and further guidance recommends that all physical therapists be equipped to manage mental health challenges common with LBP.^52^ Additionally, a recent European briefing paper on harnessing physical therapist skills for mental health solutions^53^ endorses the role of all physical therapists in screening for common mental health conditions, including depression. These documents represent critical steps towards clarifying the scope of practice and decision-making responsibilities of physical therapists. Nonetheless, clearer direction is needed in condition-specific policies and frameworks, particularly those relating to LBP. Aligned with established yellow flag and red flag screening frameworks, physical therapists require clear, practical guidance on directly screening for depression and how outcomes should inform decision-making. Evidence-based guidelines applicable across settings will support confident integration of routine depression screening into physical therapist practice.

Resource limitations within the health care system further compounded personal discomfort, contributing to participants’ reluctance to screen directly for depression. Lack of standardized referral pathways and concerns about under-resourced community services were prominent, echoing previous research highlighting physical therapists’ desire for accessible support services when managing psychological distress.^43^ System challenges were exacerbated by limited awareness of available resources and uncertainty regarding referral acceptance. Consequently, primary care physicians became the default referral option, though participants were reluctant to overburden them, reflecting an unclear understanding about their central role in managing depression in the absence of severe psychiatric symptoms or acute suicidal ideation. To support depression screening by MSK triage physical therapists, community mental health services must be adequately resourced, with clearly defined referral pathways, and improved awareness of local and regional supports. Given that screening alone does not improve outcomes,^54^ accessible and coordinated follow-up mental health services is required. Meaningful investment towards these underfunded and overwhelmed^55–57^ services is essential to ensure that depression screening translates into improved patient outcomes.

Clinic culture reinforced these personal, professional, and system capacity challenges, with time pressures and competing priorities making depression screening difficult in brief triage appointments. Consistent with existing research on psychosocial screening barriers,^12^^,^^58^ participants identified time constraints as a primary reason for prioritizing physical assessment over depression screening, with fast–paced triage appointments linked to waiting lists and discharge targets. When clinicians do allocate time to address the human aspects of care, consultations can extend up to 50 minutes, well beyond the standard 30-minute allocation.^59^ While this study was conducted within Ireland’s public health care system, appointment scheduling decisions are universally complex across health care contexts, where time and funding constraints often conflict with patient needs.^60^^,^^61^ Time pressures within clinical encounters, shaped by broader policy and funding structures, can limit opportunities for meaningful psychosocial engagement, including depression screening. Therefore, implementing routine screening in LBP care requires more than screening tools, professional training, and adequate referral resources; it also necessitates realistic scheduling models that allow holistic, patient-centered care.

Low confidence and knowledge gaps among physical therapists may unintentionally reinforce stigma.^50^ Stigma, captured under the subtheme of societal culture challenges in this study, strongly correlates with non-disclosure and topic avoidance.^62^ Participants often framed their reluctance to screen as an act of respecting patient privacy, describing a perceived discomfort on the part of the person with LBP. This perception, possibly driven by stigma, appeared to amplify depression screening hesitation. Normalizing depression screening, similar to how MSK red flag screening is normalized, may help to reduce stigma and build confidence. Without directly addressing the role of stigma, implementation efforts to improve depression screening by MSK triage physical therapists for people with LBP are likely to fall short.

There was a tendency among participants to “beat around the bush” rather than use direct screening tools or straightforward lines of questioning. This indirect, circuitousness approach appeared to function as a protective mechanism, shaped by personal discomfort, limited professional confidence, and inadequate system support. Additional deterrents to direct screening included the time constraints of triage clinics and the influence of societal stigma, which shaped participants’ belief that patients are reluctant to discuss depression when they had presented with LBP as their physical health issue. Brief screening using direct verbal questions or validated self-report tools^63–67^ may help reduce circuitous communication while remaining time efficient. A commonly recommended strategy involves initial verbal screening^67^ or a short tool such as the Patient Health Questionnaire (PHQ)-2, followed by the PHQ-9 as required, with onward referral when indicated.^68–71^ Pragmatically, busy clinicians can approach initial depression screening discreetly by integrating the questions relating to the PHQ-2 into the subjective examination using natural conversational language,^72^ following up with the PHQ-9 if clinical judgment suggests a score of ≥1.^72^^,^^73^ While such strategies and tools are useful for depression screening, identifying and responding to suicidal ideation requires particular care.^16^^,^^47^ In those instances where a person discloses suicidal ideation, a patient-centered approach with open dialog and collaborative safety planning is required.^74^

Overall, participants envisaged a culture shift in which depression screening becomes a normalized component of LBP assessment, alongside standard MSK red flag screening. Achieving this will require the coordinated implementation of brief, validated screening tools, structured referral pathways, targeted training that addresses both technical and emotional challenges, and investment in flexible scheduling and accessible mental health services. While policy developments in physical therapy and mental health are gaining momentum, this progress must be reinforced by LBP specific frameworks and guidelines that clarify professional roles and embed screening within standard care. Multi-pronged implementation efforts will be required to address the capacity, culture, and circuitous communication challenges identified in this study, and to embed effective and patient-centered depression screening as a standard component of LBP care.

### Recommendations for Future Research

Future research should investigate whether the challenges identified in this study also occur in other health care disciplines. Interdisciplinary research should develop integrated implementation strategies to address these collaboratively. Patient perspectives should be explored to provide insight into how people with LBP perceive depression screening by physical therapists, while focus groups could aid in the co-design of relevant training. Studies informed by knowledge translation frameworks should assess the acceptability and feasibility of proposed interventions, employ Delphi methodology to engage stakeholders in education and policy reform, and evaluate the long-term impact of training and policy initiatives.

### Limitations

The interviewer’s strong interest in depression screening and belief in its importance, may have influenced question phrasing or response interpretation. Similarly, the assumption that physical therapists are well-positioned to screen for depression could have shaped the interview guide. However, the interviewer’s clinical expertise and genuine engagement with the topic also enhanced the depth and relevance of the inquiry, facilitating a nuanced exploration of complex clinical perspectives.

Due to the small peer professional network, the interviewer was known to most participants, which may have led to socially desirable responding. Conversely, this familiarity fostered rapport and trust, enabling participant-centered interviews that accommodated varied communication styles and generated rich data. While some interviews were shorter than typical for in-depth qualitative research, reflecting participants’ time constraints and the focused interview guide, the experienced MSK triage physical therapists provided succinct, reflective, and relevant responses.

The study’s focus on a single country introduces sampling bias and may limit generalizability. Additionally, MSK triage physical therapists with a pre-existing interest in depression screening are likely overrepresented, and almost all had undertaken some related training.

## CONCLUSION

This study highlights the complex interplay of capacity limitations, cultural factors, and indirect communication practices that influence how MSK triage physical therapists approach depression screening for people with LBP. By drawing on the expertise of MSK triage physical therapists as subject-matter experts, the findings lay a critical foundation for implementation research and system-level change. The unanimous call for targeted training must be matched by health system investment in flexible appointment scheduling, adequately resourced community mental health services, and a supportive health care culture that normalizes depression screening as a routine, non-stigmatized component of LBP care. Ultimately, sustainable change will require coordinated policy reform to embed depression screening within standard LBP care pathways.

## Supplementary Material

PTJ-2025-0035_R2_Supplementary_Material_1_pdf_pzaf153

PTJ-2025-0035_R2_Supplementary_Material_2_pdf_pzaf153

PTJ-2025-0035_R2_Supplementary_Material_3_pdf_pzaf153

PTJ-2025-0035_R2_Supplementary_Material_4_pdf_pzaf153

PTJ-2025-0035_R2_Supplementary_Material_5_pdf_pzaf153

## Data Availability

The qualitative data generated and analyzed during this study are not publicly available due to ethical and confidentiality considerations.
