# Supplementary material for: “It’s an Uncomfortable Subject”—a Qualitative Exploration of the Challenges and Potential Solutions to Depression Screening in Low Back Pain
Source: Phys Ther. 2026 Jan 7;106(1):pzaf153. doi: 10.1093/ptj/pzaf153 (PMC12856662; doi:10.1093/ptj/pzaf153)
Supplement: PTJ-2025-0035_R2_Supplementary_Material_2_pdf_pzaf153 [file ptj-2025-0035_r2_supplementary_material_2_pdf_pzaf153.docx]

**Supplementary Material 2**

**Interview Guide for Semi-Structured Interviews**

**WARM UP**

1. Background
   - Tell me a little about your work
   - What is your experience of screening for depression in people with LBP
   - How comfortable do you feel when people with LBP tell you about their depression or suicidal thoughts?

**SCREENING**

1. Signs and symptoms/self-disclosure/questioning

- What signs and symptoms do you observe that makes you think a person with LBP might be struggling with depression?
- Do you think self-disclosure of depression without direct questioning or questionnaires is accurate?
- Do you tend to use your clinical impression or ask people directly if they are depressed?
  - 1. Why/why not?
    2. Is it difficult for you to ask people directly if they are depressed?

1. Previous training/perceived needs

- Can you describe any training which has helped to prepare to screen for depression?
- If yes, what particular training helped?
- If not, what additional training or support do you feel would be helpful?
- What do you think is needed to improve depression screening?

1. Barriers to screening

- What challenges or barriers have you noticed that makes it harder to screen for depression
- Are these unique to your work setting or generalizable?

1. Facilitators to screening

- Are there any supporting factors or enablers that might make it easier to screen for depression?
- Are these unique to your work setting or generalizable?

1. Additional tools/resources/strategies that may help the screening process

- What would might help you consistency screen people with LBP for depression?
- Are your current resources/time sufficient to do this?
- What challenges do you expect
- What would give you the confidence to implement screening

**ONWARD REFERRAL**

1. Describe your previous experience with onward referral of people with LBP and Depression

- How do you collaborate with other healthcare professionals such as psychologists or psychiatrists when managing people with LBP and depression?
- Are there standardized referral pathways/are referrals accepted from you?

1. Barriers to onward referral

- What challenges have you noticed that make the onward referral process harder?

1. Facilitators of onward referral

- Are there any factors that make it easier?

1. Additional tools/resources/strategies that help the onward referral of positive screening

- Please share any reflection on what you feel is needed/that might help the onward referral process

**END OF INTERVIEW**

- - Any additional thoughts you’d like to share?
