# Supplementary material for: “It’s an Uncomfortable Subject”—a Qualitative Exploration of the Challenges and Potential Solutions to Depression Screening in Low Back Pain
Source: Phys Ther. 2026 Jan 7;106(1):pzaf153. doi: 10.1093/ptj/pzaf153 (PMC12856662; doi:10.1093/ptj/pzaf153)
Supplement: PTJ-2025-0035_R2_Supplementary_Material_3_pdf_pzaf153 [file ptj-2025-0035_r2_supplementary_material_3_pdf_pzaf153.docx]

**Supplementary Material 3**

**Reflexive Thematic Analysis Phases, Process, and Outcomes ^1-3^**

| **RTA Phases** | **Process** | **Outcome** |
| --- | --- | --- |
| Phase One: Familiarization with the data | Active listening to audio recordings  Manual corrections of transcripts.  Transcripts read several times for familiarization  Memos and annotations logged to track initial thoughts and contextual observations.  Initial coding ideas were noted | Finalized Transcripts  Initial Memos  Initial Annotations  Initial patterns and meaningful responses and potential coding schemes |
| Phase Two: Generating initial codes | Entire dataset thoroughly and systematically reviewed, with line-by-line coding of data items deemed relevant to the research question.  Units of text dealing with the same issue were grouped together into descriptive codes.  NVivo 14 software used to organize data | Brief descriptive codes to organize similar data items from each of the 14 interviews.  29 early data items after initial coding. |
| Phase three: Generating themes | Initial codes were reviewed, and related codes were grouped, integrated, and analyzed according to shared meaning so that focus shifted toward the construction of themes and subthemes of meaning across the dataset.  NVivo 14 software used to organize data | During initial stages of phase 3 there were 8 categories/themes |
| Phase four: Reviewing potential themes | Iterative process of consolidation of themes and sub themes was carried out, with multiple recursive reviews and refinements of themes and sub themes to ensure data items and codes within each theme were appropriate, and to analyze whether themes accurately represented the data and pertained to the research question.  Some themes were merged, some were split, and some were abandoned.  Both NVivo software and manual coding framework were used in addition to visual mapping. | A richer understanding of the data, which was far removed from the earlier simplistic and descriptive codes.  Multiple iterations of coding revision to facilitate meaningful reinterpretations of the data in relation to the research question  Development of an end stage thematic map. |
| Phase five: defining and naming themes | Themes and sub themes were revised to ensure their nomenclature and content were internally consistent, with illustrative extracts chosen to convey meaning in the context of the research question. | Three final themes relating to challenges are presented in Figure 1, Table 2 and Supplementary Material 4; capacity, culture, and circuitousness*.* Capacity had three subthemes; personal capacity*,* professional capacity*,* and system capacity. Culture had two subthemes; clinic culture and societal culture.  Five final themes relating to solutions are presented in Figure 2 and Supplementary Material 5; screening tools, normalizing screening, training, pathways, and resources. |
| Phase six: producing the report | A summary of synthesized themes relating to the research question, in the context of available literature, was produced. | |

References

1. Braun V, Clarke V. Using thematic analysis in psychology. *Qual Res Psych*. 2006;3(2):77-101. doi:10.1191/1478088706qp063oa

2. Braun V, Clarke V. Reflecting on reflexive thematic analysis. *Qual Res Sport Exerc Health*. 2019;11(4):589-597. doi/10.1080/2159676X.2019.1628806

3. Braun V, Clarke V. Can I use TA? Should I use TA? Should I not use TA? Comparing reflexive thematic analysis and other pattern‐based qualitative analytic approaches. *Couns. Psychother. Res*. 2021;21(1):37-47. doi:10.1002/capr.12360
