# Supplementary material for: “It’s an Uncomfortable Subject”—a Qualitative Exploration of the Challenges and Potential Solutions to Depression Screening in Low Back Pain
Source: Phys Ther. 2026 Jan 7;106(1):pzaf153. doi: 10.1093/ptj/pzaf153 (PMC12856662; doi:10.1093/ptj/pzaf153)
Supplement: PTJ-2025-0035_R2_Supplementary_Material_4_pdf_pzaf153 [file ptj-2025-0035_r2_supplementary_material_4_pdf_pzaf153.docx]

**Supplementary Material 4**

**Illustrative Participant Quotations: Challenges to Depression Screening in MSK Triage**

| **Capacity “*How Are You Going to Deal With [It]”*** | |
| --- | --- |
| **Personal Capacity**  ***“Out of Your Comfort Zone”*** | *“I don't have the capacity… to go into that”* (Alice)  *“is not something I would feel is appropriate to be delved into”* (John)  *“is it appropriate for a physio to be screening?”* (John)  *“There's a degree of impostor syndrome”* (James)  *“it's an uncomfortable subject to broach with people”* (James)  *“what probably scares people is that they don't want to kick the rock over because God knows what they'll find”* (James)  *“it is scary…you're asking him, like, "Do you have a plan", like, that's terrifying”* (James)  *“there's that fear aspect of…allowing somebody to be upset”* (Daniel)  *“I think any discomfort I felt in the past was more related to me necessarily than the patients”* (Daniel)  *“I'd be very reticent to ask about it”* (Grace)  *“knowing that they're under the right care when you finish work at 4:30 here or whatever, that would be a big worry, a big concern”* (Lisa)  *“when it comes into like more acute kind of mental health, obviously you get a little bit more less comfortable because it's getting a bit beyond your scope in terms of then”* (Leah)  *“not being confident in my own ability, to assess the risk really”* (Leah) |
| **Professional Capacity**  ***“I Don't Feel Like I Have the Training”*** | *“I have no training and I don’t know where to send them”* (David)  *“taking on board something that you're not trained to deal with”* (John)  *“I don't have the training in it or the expertise in it and that's why it can sometimes be difficult”* (John)  *“I don't know if I if I did try to refer someone to the community for psychology, would they accept it? I actually don't know that”* (Anna)  *“knowing…what to do with that information is harder than the asking of us”* (Emily)  *“the hard part is knowing where to direct patients….I don't feel I have the expertise or the knowledge to know where the most appropriate person or the most appropriate place to start care is”* (Emily)  *“I would have a lack of awareness as to what, for example, counselling services can offer”* (Emily)  *“I wouldn't be aware of the inclusion/exclusion criteria for their own services and what they feel that they can offer”* (Emily)  *”I don't actually know what's kind of available out there. Yeah. So generally, I would, I would kind of land it back on the GP”* (Sophie)  *“who would I refer them on to”* (Grace)  *“I don’t know…where to put them”* (Grace)  *“I don’t know the right question to ask”* (Grace)  *“what do I do with that information”* (Emma)  *“I wouldn't know what is acceptable to even start to refer in….I wouldn't know who I can refer or why I should refer”* (Emma)  *“I don't know if we can refer directly into mental health. I presume we can't”* (Emma)  *“you have vague idea, but you'd love to have a definite this is what I do or this is where I turn to”* (Kate)  *“It's…out of your kind of realms of professional, I suppose capacity as well”* (Kate)  *“what other services are out there that's going to be of benefit to the patient and help them to manage their symptoms”* (Lisa)  *“there is people to contact, it's just that people don't know who they are”* (Leah)  *“So it's probably not asking the question but deciding”* (Leah)  *“What else should I be asking or how do I interpret those answers? I don't know. You know, sometimes it's difficult”* (Leah)  *“ Where my limitations would lie would be like that on what to act on or how to act on it or having direct links with you know professionals in the hospital …you know I wouldn't maybe necessarily have links with people with whom I could you know explore those further or refer patients on to I wouldn't be that aware of where my you know my referral rights or my links would be with”* *(Michael)* |
| **System Capacity**  **“The Resources Just Aren’t There*”*** | *“if you are sending a patient to somewhere where they're going to sit on a list somewhere else. It's not very good practice”* (Alice)  *“And like it is that you do refer on to the GP, hoping that there's a referral, but knowing that the system's probably not capable of handling it”* (James)  *“They don't cross a threshold to be sent to psychiatric services so they're kind of back in limbo again”* (James)  *“we're kind of left carrying the can”* (James)  *“I wouldn't have anywhere to go with the patient”* (Grace)  *“you just want to feel that you have back up”* (Kate)  *“I suppose knowing where to refer, who to refer to, how to refer. Yeah, not having a definite onward referral system I think is part of the problem. At the moment you have vague idea, but you'd love to have a definite this is what I do, or this is where I turn to, this is where I look to for help”* (Kate)  *“If they go back to the GP, they then just get referred back in and they're put on the back another waiting list”* (Michael) |
| **Culture *“It's Difficult”*** | |
| **Clinic Culture**  **H*huge Time Pressure*”** | *“And what the way the clinics and stuff are set up, we don't have the time” (John)*  *“it's not something that's going to be managed within 15 to 20 minute consultation”* (John)  *“depending on the clinic and how much time you have, this and what else has happened in that day will influence how much time you have to go into that discussion”* (Emily)  *“varies depending on how much pressure I am on under the on the day” (Sophie)*  *“it's difficult in a triage quick appointment”* (Emma)  *“Time is one I suppose depending on your day. Do you know what I mean…in the middle of a busy clinic or running over on time, that is definitely going to be a barrier*” (Kate)  *“you do have to think about the time efficiency of it as well…in an ideal world you have all the time to explore it, delve into it. But we don't”* (Kate) |
| **Societal Culture**  ***“I Don’t Want to Make the Patient Uncomfortable”*** | *“there's a reticence on the patient's part of bringing it up”* (James)  *“**I think they're not expecting it, you know.” (Sophie)*  *“Is the patient comfortable having that discussion” (Kate)*  *“I think it's a very personal area you're delving into. Do you know what I mean? So there's always going to be a little bit of discomfort there”* (Kate) |
| **Circuitousness “B*eating Around the Bush”*** | |
| *“I probably more so react going with prompts from the patient” (David)*  *“Being asked sensitively, like, are you depressed is probably not the or it may be the best way to, I don't know”* (James)  *“I still don't actually know how to engage somebody. I might just ask them, you know, I might, my go to, I might acknowledge the pain is physically mental, emotional, spiritual. Maybe the hope that they'll, you know” (Daniel)*  *“It's not something I would ever ask. Unless the patient….opens the conversation, and so it might be that sometimes they feel that life isn't worth living with this amount of pain. You know that you can, you have a way in then” (Emily)*  *“you're kind of waiting to see if anything comes from them and then you're seeing can you kind of probe a little bit and ideally, if they lead us a little bit and you can kind of jump on the bandwagon” (Sophie)*  *“I don't know if I'd ever ask someone “do you have depression” or “do you suffer from mood disorder” (Grace)*  *“if they don't tell me they have it, I I'm probably slow to ask them about it” (Grace)*  *“It's more something that I feel comes up with over your conversation with just during your general subjective” (Kate)*  *“generally aggravating and easing factors as my opener or my opening into understanding if there's a mental health component to it” (Lisa)*  *“I suppose my kind of approach is that I've kind of pre, pre prefaced it with like the impact of pain on the all the very different areas of life” (Leah)*  *“you'll maybe just skirt around it” (Michael)* | |
