# Supplementary material for: “It’s an Uncomfortable Subject”—a Qualitative Exploration of the Challenges and Potential Solutions to Depression Screening in Low Back Pain
Source: Phys Ther. 2026 Jan 7;106(1):pzaf153. doi: 10.1093/ptj/pzaf153 (PMC12856662; doi:10.1093/ptj/pzaf153)
Supplement: PTJ-2025-0035_R2_Supplementary_Material_5_pdf_pzaf153 [file ptj-2025-0035_r2_supplementary_material_5_pdf_pzaf153.docx]

**Supplementary Material 5**

**Illustrative Participant Quotations: Potential Solutions to Depression Screening in MSK Triage**

| **Training and Education *“To Know When to Raise the Flag”*** |
| --- |
| *“Everybody should be trained to a basic clinical level and to know when to raise the flag…..it nearly, it nearly just falls into safeguarding, doesn't it? Like it should be done nearly along with your manual handling and your BLS”* (Alice)  *“There should be a quick and short HSELand module that's no more than an hour”* (Alice)  *“So somebody with seniority who's been trained to an advanced practise level should be adequately able to screen for it at least”* (John)  *“You should be able to ask the questions and have an idea what to do with the given response”* (John)  *“I think the answer has to be that everybody's brought up to a standard with training”* (James)  *“training so that you're comfortable, you can identify it”* (James)  *“There's nothing there, so anything is better than nothing, so something simple that is accessible for everyone that gives people enough to go. OK, I'm comfortable with this, that I'll actually ask a patient to start with, that gets a conversation going”* (James)  *“it needs to be face to face, it needs that that personal approach, because I don't think you would you would allow yourself the time to reflect on HSELand, because we all know it's a it's a tick box, it’s how am I going to answer my last 6-8 questions”* (Daniel)  *“I don't think it takes a huge amount of training to have an awareness of it”* (Anna)  *“I think education is really important”* (Emily)  *“Education and courses, but also I think that pure learning and being open to the expertise that's around you and tapping in tapping into that”* (Emily)  *“I think be helpful to hear from people outside of physiotherapy* *that, that that, that psychological area or depression is their area of expertise”* (Emily)  *“I think yes, training will probably be welcome. I mean that possibly could be something online”* (Sophie)  *“a pre-recorded webinar by somebody with a bit of expertise in this”* (Sophie)  *I think I'd like a daylong face to face course”* (Grace)  *“I think if I was educated in what to ask or why to ask like, what's the benefit of asking? How will that change my treatment? I think I would”* (Grace)  *“I would like some training in, maybe the flags, to recognise the flags that would say the potentially they have some sort of psychological disorder that is affecting their condition and what to ask then”* (Grace)  *“Yeah with probably different sorts of health professionals. Like maybe a psychiatrist, psychologist, a psychotherapist. You know, different people. Maybe even a patient or two, you know”* (Grace)  *“ I think webinars are a waste of time. An online course in HSELand is a waste of time. I think I'd like a daylong face to face course”* (Grace)  *“learning from other people's yeah experience in the area”* (Kate)  *“kind of from a learning from each other. Learning from our experiences as well as any formal”* (Kate)  *“Ideally probably shorter, more succinctly, it would be better just even from ease of access and just being able to take the time to do it”* (Kate)  *“I do think probably face to face will work better because I do think a lot of it would depend on interactions and people's experiences which I think you just wouldn't be able to avail of as easily on an online forum”* (Kate)  *“a one-to-one training day you get all that information, but actually you haven't had a chance to then try and apply any of the clinically and to feedback on it. Or so I think a series of webinars is useful”* (Lisa)  *“I would no doubt have training needs in that area”* (Michael)  *“a baseline level of training might help. And, whether that's something at a local level because that's always going to be different than whichever service that you work in”* (Michael)  *“if it was you know it was to be a day thing then I think face to face is always a little bit better because there's opportunity for you know discussion between people, breakout sessions on Webex or zoom or teams are never really the same”* (Michael) |
| ***Standardized Pathways “There's a Cauda Equina Pathway. Why Wouldn't There Be, Like, a Mental Health First Aid One”*** |
| *“I think having really clear pathways. I know there's a lot of work going on with modernised care pathways in the HSE at the moment, but I think for all therapists to understand the pathways that are available, if we could get the patient down the right path*” (Alice)  *“I appreciate the pathways differ around the country, but…that would be a good start”* (Alice)  *“I suppose having that back-end piece where it's like if someone does talk about X, Y, or Z this is the appropriate pathway that you can put them on”* (James)  *“in case of emergency break glass that that kind of sort of”* (James)  *“There obviously needs to be a pathway, doesn't there? Because, you know, we we're dealing with people with chronic pain, which we know will have to affect their mental, emotional health”* (Daniel)  *“I suppose in a way, the very unwell, that kind of almost crisis intervention is a little bit more straightforward to some degree”* (Sophie)  *“I suppose if they're if they're that far down the road and they're expressing suicidal ideation or plans like you are, you're going to send them to ED or contact whoever the liaison psychiatrist in ED is. That's probably a simpler pathway. I know what to do in that case. It's all the stuff before it that I don't know what to do it”* (Grace)  *“if there are pathways set up….that’s probably the most important part”* (Grace)  *“if you knew that there was a pathway that you could refer into, I think that would maybe make me more open to open asking questions”* (Emma)  *“But it was easier to feel that you had done all that you could. Like. That's OK. I have flagged this, but next thing I've flagged it with the team and they've been referred to psychiatry”* (Kate)  *“I think I think like a clear a clear pathway like I went out of my way to identify like who the person is I need to call to, like, find out if this scenario happens, what action I need to take”* (Leah)  *“Whose role is it? The Reg? Do you call The Reg? Is it The Reg that calls this psychiatric liaison nurse? Where do you send the patient? Do they phone them like it's all a bit vague”* (Leah)  *“So, like, there's a cauda equina pathway. Why wouldn't there be, like, a mental health first aid one”* (Leah)  *“For me, what would look best for me would be a pathway because that's, you know. Well, that's the way my mind works”* (Michael)  *“the best pathway is not necessarily back to the GP”* (Michael)  *“you contact on call psychiatry, which is my understanding, and you don't let you don't leave the room, you know, if you do have to leave the room to make phone calls, you get something sit with them or whatever. I'm comfortable enough with that”* (Michael) |
| **Knowledge of and Access to Resources *“Knowing Where to Refer, Who to Refer to, and How to Refer”*** |
| *“if you've got really good set up and support….., then you know you can open that can of worms with the patient and you've somewhere to send them where they'd be better managed”* (Alice)  *“The problem is access, like where do we send them… a change in access would help*” (David)  *“I also need links to psychology counsellor and I suspect they are also under resourced and under pressure”* (David)  *“just to know where to send him on what the avenues are”* (Alice)  *“knowledge of the local psychiatric services, et cetera for severe cases”* (John)  *“I suppose my first protocol generally is the GP”* (James)  *“Where do you send them? They don't cross a threshold to be sent to psychiatric services so. They're kind of back in limbo again”* (James)  *“it would certainly be helpful to know more about the types of patients that they feel are appropriate”* (Emily)  *“I don't think we have a problem asking the questions, but we have a problem with knowing what to do with the answer and if we had that knowledge”* (Emma)  *“I suppose knowing where to refer, who to refer to, and how to refer. Yeah, not having a definite onward referral system I think is part of the problem at the moment you have vague idea, but you'd love to have a definite this is what I do or this is where I turn to. This is where I look to for help”* (Kate)  *“I don't have any direct referral access into other services, so I will put it into the GP letter and ask as one of the recommendations from clinic that the GP reviews their mental health”* (Lisa) |
| **Screening Tools “*That Get a Conversation Going”*** |
| *“something simple that is accessible for everyone…* *that get a conversation going”* (James)  *“short and simple with some kind of reasonable kind of sensitivity to pick these things up”* (Sophie)  *“a validated kind of one line question that…would just automatically become part of you know you would screen”* (Sophie)  *“I'm not against the questionnaires if it’s short and not vague”* (Grace)  *“a quick and easy one…in an ideal world that you have all the time to explore it, delve into it. But we don't like so”* (Kate)  *“a tool that you can either formally do with the patient or that you can kind of nearly mark off yourself based on that conversation thinking, OK, I have an idea now what threshold they're at, what do I need? And you know what you need to do then that would be good”* (Kate) |
| **Normalize Screening *“No Different to Screening for Red Flag Pathology”*** |
| *“it should be stuff that you able to ask no different to screening for red flag pathology and sinister pathology”* (John)  *“I think the more you ask about it, the easier it gets to ask”* (John)  *“if you have a system in place that as part of your standardized objective, it gets asked regardless”* (James)  *“it's like we ask him about red flags. Every single person gets asked that stuff”* (James)  *“I think it needs to be as standard as your physical assessment”* (Anna)  *“I try to make it a fairly natural part of my kind of subjective screening now and just as much as I asked him about their physical well-being”* (Anna)  *“the more you do it, the easier it gets and if you just make it a normal part of your process then, you know the worst that happens is they say I'm fine*” (Anna)  *“I will ask the question quite frequently, I'll be quite direct, and I frequently use it as part of my medical history”* (Emily)  *“surely that should be standardized as just your musculoskeletal assessment”* (Leah) |
